# Anti-*Trypanosoma cruzi* Activity of Extracts from Argentinean Asteraceae Species

**DOI:** 10.22037/ijpr.2019.14491.12430

**Published:** 2019

**Authors:** Mariana G. Selener, Orlando Elso, Carla Grosso, Jimena Borgo, María Clavin, Emilio L. Malchiodi, Silvia I. Cazorla, Flavia Flavia, Valeria P. Sülsen

**Affiliations:** a *Cátedra de Farmacognosia, Facultad de Farmacia y Bioquímica, Universidad de Buenos Aires, Buenos Aires, Argentina. *; b *Instituto de Química y Metabolismo del Fármaco (IQUIMEFA) (UBA-CONICET), Buenos Aires, Argentina. *; c *Cátedra de Inmunología, Instituto de Estudios de la Inmunidad Humoral (IDEHU) (UBA-CONICET), Facultad de Farmacia y Bioquímica, Universidad de Buenos Aires, Buenos Aires, Argentina. *; d *Instituto de Microbiología y Parasitología Médica (IMPaM) (UBA-CONICET), Facultad de Medicina, Buenos Aires, Argentina.*; e *Centro de Referencia para Lactobacilos (CERELA) (CONICET), San Miguel de Tucumán, Argentina.*; 1 *F. Redko and V. Sülsen contributed equally to this work.*

## Abstract

The anti-*Trypanosoma cruzi* activity of extracts from 13 Argentinean Asteraceae species was determined. Dichloromethane and methanol extracts of *Acmella bellidioides*, *Aspilia silphioides, Viguiera tuberosa, Calyptocarpus biaristatus, Hyalis argentea, Helenium radiatum, Gaillardia megapotamica, Verbesina subcordata, Gymnocoronis spilanthoides, Viguiera anchusaefolia, Thelesperma megapotamicum, Zexmenia buphtalmiflora*, and* Vernonia plantaginoides *were evaluated *in-vitro* against* Trypanosoma cruzi *epimastigotes*. A. silphioides*, *V. tuberosa*, *V. subcordata*, *G. spilanthoides*, *G. megapotamica*, *T. megapotamicum *and *Z. buphtalmiflora* dichloromethane extracts showed trypanocidal activity with inhibitions higher than 60% at a concentration of 10 µg/mL. The methanol extracts of *H. radiatum *and *G. megapotamica *were the most active with inhibitions of 70.1 and 77.7%, respectively at 10 µg/mL. The chromatographic profiles of the most active extracts showed bands and major peaks that could be attributed to flavonoids and terpenoid compounds.

## Introduction

Chagas’ disease or American Trypanosomiasis, caused by the protozoan parasite *Trypanosoma cruzi*, constitutes a serious health problem mainly in Latin America. It is trasmitted by triatomine bugs (insect vector) that carry the parasite. According to the World Health Organization, this parasitosis affects 6-7 millions people worldwide (1). Due to migration phenomena, this disease has now spread to developed countries such as the EEUU, Canada, Japan, Australia and some European countries, where the transmission is produced mainly through non-vectorial routes (2). 

In Argentina, it is the most relevant protozoan parasitic disease affecting approximately 1.5 million people (3). Chagas’ disease treatment is limited to two drugs, nifurtimox and benznidazole, that are effective mainly during the acute stage of the disease and have severe side effects. The side effects and the emerging resistance to available drugs have led to an urgent need for new therapeutic agents. 

Natural products have provided useful drugs that are used nowadays to treat different diseases: digoxin, morphine, vincristine, vinblastine, taxol, etc., among others. With regards to antiparasitic molecules of natural origin, quinine and artemisinin and their derivatives are used along or in combination therapies to treat malaria. Artemisinin is a sesquiterpene lactone isolated from the chinese medicinal plant *Artemisia annua* (Asteraceae) that has been used to treat fevers asociated with malaria (4). 

Asteraceae is one of the largest families in the world with approximately 24000-30000 species (5). In Argentina, Asteraceae (or Compositae) constitute the most numerous and diverse important vascular plant family, comprising 223 genera and about 1500 species (6). Asteraceae species have been a rich source of active compounds and have been attractive for drug discovey since they are higly promising from a pharmacological perspective. 

In this sense, the aim of the present study was to determine the anti-*Trypanosoma cruzi *activity of Argentinean Asteraceae species in the search of novel trypanocidal lead compounds.

## Experimental

*Plant material*

Plant material (aerial parts) was identified by Dr. Gustavo Giberti and voucher specimens were deposited at the Museo de Farmacobotánica, Facultad de Farmacia y Bioquímica, Universidad de Buenos Aires. 

The selected species, the identification numbers and place and date of collection are shown in Table 1.

*Parasites*

*Trypanosoma cruzi* epimastigotes República Argentina (RA) strain were grown at 28 ºC in liver infusion tryptose (LIT) medium supplemented with 10% foetal bovine serum. The cultures were routinely maintained by weekly passages at 28 °C. 

*Preparation of extracts*

Dried plant material (20 g) was extracted with dichloromethane (200 mL), at room temperature and then filtered. The process was repeated twice, and the filtrates were combined and taken to dryness under vacuum. The plant material was then extracted with methanol (200 mL) for 24 h, and the filtrates were combined and taken to dryness under vacuum. Dichloromethane and methanol extract yields (g of extract/100 g plant) were: *Acmella bellidioides*: 0.2 and 2.7%;* Aspilia silphioides*: 0.2 and 8.9%;* Viguiera tuberosa*: 0.3 and 8.5%;* Calyptocarpus biaristatus*: 0.3 and 3.7%;* Hyalis argentea*: 0.3 and 9.2%;* Helenium radiatum*: 3.5 and 8.1%;* Gaillardia megapotamica*: 3.2 and 7.6%;* Verbesina subcordata*: 0.4 and 2.0%;* Gymnocoronis spilanthoides: *0.2 and 3.8%; *Viguiera anchusaefolia: *0.3 and 1.5%;* Thelesperma megapotamicum*: 0.5 and 7.0%;* Zexmenia buphtalmiflora: *0.3 and 5.8%;* Vernonia plantaginoides*: 0.3 and 2.3%.

*In-vitro trypanocidal activity*

The anti-*T. cruzi *activity of the extracts was assayed on epimastigote forms using the [^3^H] thymidine uptake assay (7). Exponentially growing epimastigotes were adjusted to a cell density of 1.5 × 10^6 ^parasites/mL in fresh medium. The extracts were initially dissolved in DMSO at 400 times the desired final maximum test concentration and were diluted in fresh LIT medium (Liver Infusion Broth) at final concentrations of 10 and 100 µg/mL . The control parasites were exposed to an equivalent concentration of DMSO (0.25% v/v, negative control). 

The parasites were allowed to grow for 72 h in medium alone or in the presence of the extracts dilutions in triplicate at 28 °C. The Percentage of inhibition was calculated as {100 − [(cpm of treated parasites/ cpm of untreated parasites) × 100]}. Benznidazole was used as positive control. 

*Thin layer chromatography analysis (TLC)*

The chromatographic analyses of dichloromethane extracts of *A. silphioides* (ASDE) and *T. megapotamicum *(TMDE) were performed on Silicagel plates (F_254_, Merck) developed with Hexane:EtOAc (8:2) (system I) and dichloromethane:MeOH (95:5) (system II) and sprayed with anisaldehyde sulfuric acid reagent. Methanol extracts of *H. radiatum *(HRME) and* G. megapotamica* (GMME) were assayed on Silicagel plates (F_254_, Merck) developed with EtOAc:Toluene:MeOH:Formic Acid (60:20:10:10) (system III) and on Cellulose plates (Merck) with AcH 40% (system IV) and sprayed with Natural Products reagent (NPR). Rf values of the major bands were calculated.

*High Performance Liquid Chromatography Analysis (HPLC)*

The chromatographic HPLC profiles of ASDE, TMDE, HRME, and GMME were performed using a Waters equipment with photodiode array detector (Waters 2996), a Rheodyne injection valve (20 μL), pump (Waters Delta 600), Waters 600 controller and in-line degasser. A reversed-phase column (LiChroCART 125-2, 4 µm Supersphere 100 RP-18, Merck) was used and the photodiode array detector was set at 220 and 330 nm. ASDE and TMDE samples were eluted with a gradient of water (A) and acetonitrile (C) from 15% C to 100% C in 15 min. HRME and GMME samples were analyzed with a gradient of water/AcH 2% (A) and methanol (B) from 15% B to 100% B in 15 min. The flow rate was 1.0 mL/min and the separation was performed at room temperature. Chromatograms were recorded and processed using the Empower software. Dichloromethane and methanol extracts were dissolved in methanol to a final concentration of 10 mg/mL. The water employed to prepare the mobile phase was of ultrapure quality (Milliq). Acetonitrile (HPLC) J. T. Baker and methanol (HPLC) J. T. Baker were used.

*Statistical analysis*

Results are presented as mean ± SEM employing the GraphPad Prism 5.0 software (GraphPad Software Inc., San Diego, CA). 

The statistical significance was determined by Kruskal Wallis test performed with the GraphPad Prism 5.0 software (GraphPad Software Inc., San Diego, CA). Comparisons were referred to the control group. *p* values <0.05 were considered significant.

## Results and Discussion

Natural products have played an important role in drug discovery process. The great diversity and complexity of skeletal and structures that natural products offer is of great interest in the discovery and development of new pharmaceutical lead compounds. 

The interest in Asteraceae species in the search of new bioactive molecules has increased in the last years due to the amount and diversity of the compounds they produce. In this sense, we have selected thirteen Argentinean species and evaluated them on *T. cruzi*. Dichloromethane and methanol extracts were prepared for each plant. The trypanocidal activity of the extracts was assessed against *T. cruzi* epimastigotes. The percentage of growth inhibition of dichloromethane and methanol extracts of each species is shown in Table 2.

All dichloromethane extracts showed significant activity against epimastigotes at 100 µg/mL (> 80%). Dichloromethane extracts of *A. silphioides*, *V. tuberosa*, *V. subcordata*, *G. spilanthoides*, *G. megapotamica*, *T. megapotamicum*, and *Z. buphtalmiflora* were active against *T. cruzi *with inhibitions higher than 60% at 10 µg/mL. At this concentration, *A. silphioides* and *T. megapotamicum *dichloromethane extracts were the most active with percentages of growth inhibition of 93.4 and 95.6, respectively. Only the methanol extracts of *H. radiatum *and *G. megapotamica *have demonstrated high trypanocidal activity with inhibitions of 70.1 and 77.7%, respectively at the the lowest tested concentration. This is the first report about the anti- *T. cruzi* activity of the selected species, with the exception of *G. megapotamica* from which the active sesquiterpene lactone helenalin was isolated (8). Besides, it has been reported that the extracts of this species showed *in-vitro* cytotoxic and antioxidant activity and was effective in inhibiting the growth of *Fusarium verticillioides* (9-11). Dichloromethane and methanol extracts from two other argentine species of *Gaillardia *have also shown antiprotozoal activity (12). 

Total phenol content and antioxidant capacity and pesticide activity has been described for *G. megapotamica,* *T. megapotamicum*, and *Z. buphtalmiflora *(13, 14). The isolation of sesquiterpene lactones, a pseudoguaianolide, and a guainianolide rhamnopiranoside, and flavonoids has been described in *H. radiatum* (15). Diterpenoids, sesquiterpene lactones, diterpene lactones, and a coumarin have been isolated from *H. argentea* (16). Some of the terpenoids isolated from this species have shown antifungal activity (17). Several eudesmane derivatives were identified in *Verbesina* species including *V. subcordata *(18). The presence of pyrrolizidine alkaloids was confirmed in *G. spilanthoides*, being lycopsamine and intermedine the major ones (19). Neither chemical nor biological reports of *A. bellidiodes*, *A. silphioides*, *V. tuberosa*, *C. biaristatus*, *V. anchuseifolia*, and *V. plantaginoides* could be found in the literature.

**Figure 1:**
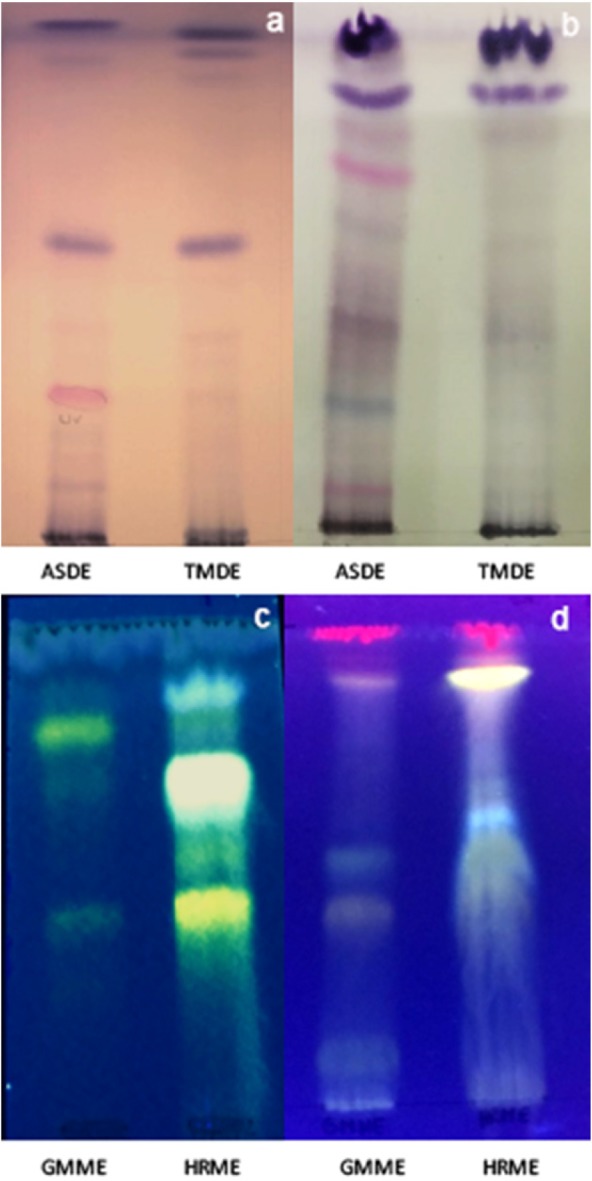
TLC chromatographic profiles of dichloromethane extracts of *A. silphioides* (ASDE) and *T. megapotamicum *(TMDE) and methanol extracts of *H. radiatum *(HRME) and *G. megapotamica* (GMME). a: system I (sprayed with anisaldehyde sulfuric acid reagent), b: system II (sprayed with anisaldehyde sulfuric acid reagent), c: system III, (sprayed with Natural Products reagent), d: system IV (sprayed with Natural Products reagent). The chromatograms were observed in visible light (a and b) and under UV light at 366 nm (c and d)

**Figure 2:**
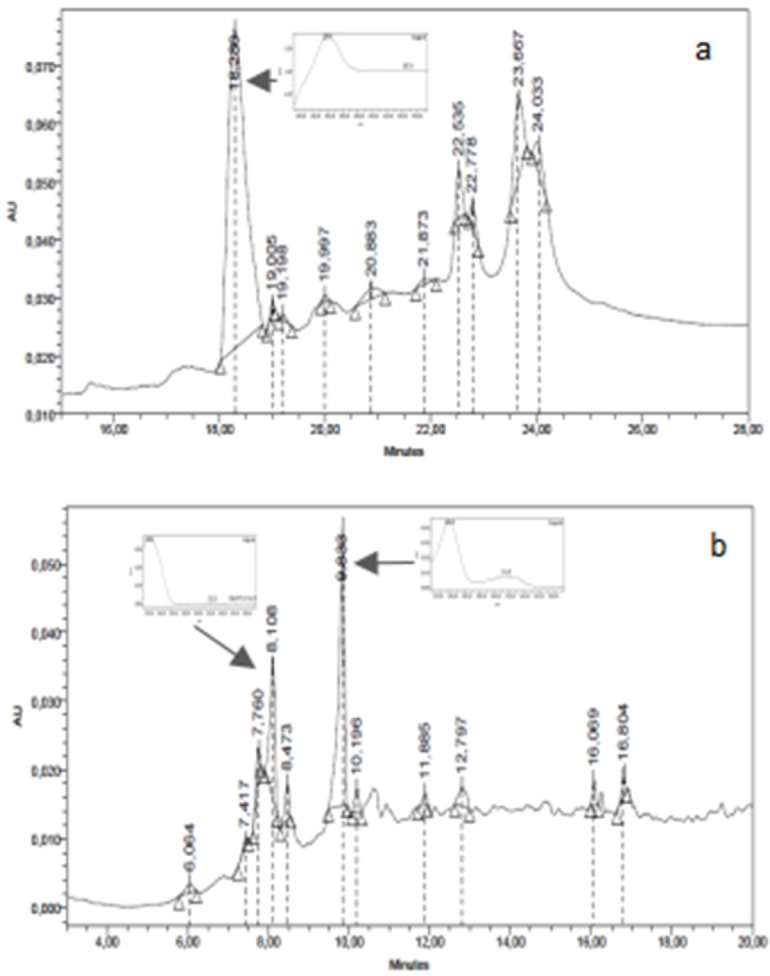
HPLC chromatographic profiles of dichloromethane extracts of *A. silphioides* (ASDE): a and *T. megapotamicum *(TMDE): b

The thin layer chromatographic profiles of ASDE showed the presence of the major bands (4 on system I and 8 on system II, colours: purple, pink, and blue). The TMDE presented 4 and 3 major bands on system I and II, respectively, giving purple spots after spraying with anisaldehyde sulfuric acid reagent (Figure 1a and Figure 1b). The HPLC profile of ASDE showed one major peak with a retention time of 18.3 min, with an UV maximum at 250 nm (Figure 2a). In the case of TMDE, the analysis of the HPLC profile showed 2 major peaks (8.1 and 9.3 min) which were detected at UV 220-230 nm (Figure 2b). These results suggest that terpenoid compounds may be present in ASDE and TMDE.

**Figure 3:**
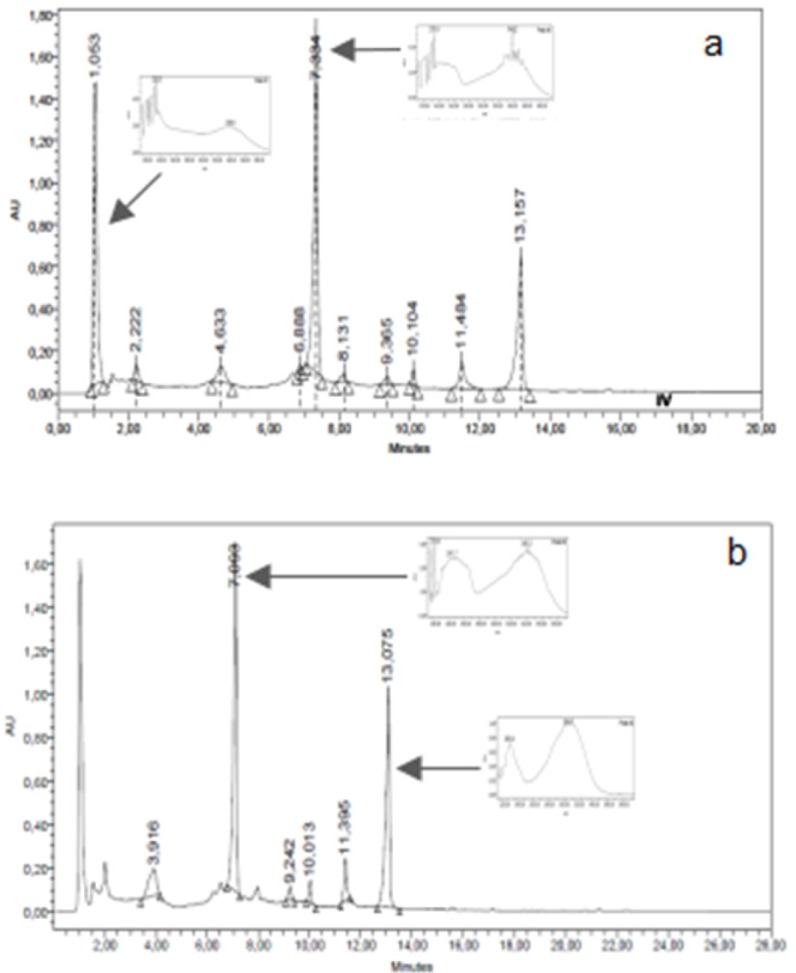
HPLC chromatographic profiles of methanol extracts of *H. radiatum *(HRME): a and *G. megapotamica* (GMME): b

The TLC chromatographic profiles of GMME on system III presented 4 yellow and orange major bands with Rf values of 0.11, 0.39, 0.43, and 0.89. On system IV, 3 yellow fluorescent bands (Rf values: 0.42, 0.69 and 0.81) were observed. With regards to HRME, after TLC analyses this extract showed 5 major fluorescent bands (yellow, light blue, orange, and green) (Figures 1c and 1d). The GMME extract presented 2 major peaks (1.1 and 7.3 min) with UV maximum at 230 and 340 nm and HRME showed 2 major peaks at 7.1 min (240 and 340 nm) and 13.1 min (226 and 306 nm) (Figures 3a and 3b). The UV spectra of the major peaks in these two active extracts are characteristic of phenolic compounds.

Anisaldehyde sulfuric acid is a universal reagent that has been used mainly for the detection of terpenes and steroids giving violet, blue, red, grey, or green spots after spraying and heating TLC plates. Natural products reagent allow the detection of phenolic compounds, which gives yellow, orange, green/light blue fluorescence zones in UV-366 nm. 

The economical and medicinal importance of Asteraceae has been widely described (20). Chemically, a variety of the compounds have been isolated from these species being terpenes and phenolic compounds among the most relevant. There are previous reports about the antiprotozoal activity of Asteraceae species and their constituents (21-23). Several terpenoid compounds such as sesquiterpene lactones and diterpenes, and flavonoids, isolated from plants of this family, have shown significant antiprotozoal activity against different parasites including *Trypanosoma cruzi* (7, 23-27). The trypanocidal activity of the methanol extract of *H. radiatum *could be due to the presence of flavonoids and glycosylated sesquiterpene lactones since this type of compounds has been previously reported in this species (15). Terpenoids and phenolic compounds such as flavonoids, which have been detected in the dichloromethane extracts of *A. silphioides*, *T. megapotamicum, *and *G. megapotamica* methanol extract, among others constituents could be responsible for the observed anti-*T.cruzi* activity.

**Table 1:** Argentinean Asteraceae species screened against *Trypanosoma cruzi*

**Species**	**Identification number**	**Place of collection**	**Date of collection**
*Acmella bellidioides *(Sm.) R.K. Jansen	BAF 736	Entre Rios Province, Argentina	November 2011
*Aspilia silphioides *(Hook. & Arn.) Benth. & Hook.	BAF 740	Entre Rios Province, Argentina	November 2011
*Viguiera tuberosa *Griseb.	BAF 731	Entre Rios Province, Argentina	November 2011
*Calyptocarpus biaristatus *(DC.) H. Rob.	BAF 737	Entre Rios Province, Argentina	November 2011
*Hyalis argentea *D. Don ex Hook. & Arn. et Arn. var. *latisquama *Cabrera	BAF 749	Buenos Aires Province, Argentina	February 2012
*Helenium radiatum *(Less) Seckt	BAF 751	Buenos Aires Province, Argentina	February 2012
*Gaillardia megapotamica *(Spreng.) Baker var. *megapotamica*	BAF 752	Buenos Aires Province, Argentina	February 2012
*Verbesina subcordata *DC.	BAF 767	Entre Rios Province, Argentina	March 2012
*Gymnocoronis spilanthoides *(Hook. & Arn.) DC. var. *subcordata *(DC.) Baker	BAF 787	Entre Rios Province, Argentina	December 2012
*Viguiera anchusaefolia *(DC.) Baker	BAF 796	Corrientes Province, Argentina	December 2012
*Thelesperma megapotamicum *(Spreng.) Kuntze	BAF 753	Buenos Aires Province, Argentina	February 2012
*Zexmenia buphtalmiflora *(Lorentz) Ariza	BAF 754	Buenos Aires Province, Argentina	February 2012
*Vernonia plantaginoides *(Less.) Hieron.	BAF 790	Corrientes Province, Argentina	December 2012

**Table 2:** *In-vit*ro trypanocidal activity of Argentinean Asteraceae species on *Trypanosoma cruzi *epimastigotes

**Species**	**% Growth inhibition**			
	**Dichloromethane extract**		**Methanol extract**	
	**100 µg/mL**	**10 µg/mL**	**100 µg/mL**	**10 µg/mL**
*Acmella bellidioides*	97.8±0.4**	54.6±4.0*	13.9±5.6	22.4±7.4
*Aspilia silphioides*	97.7±0.2**	93.4±0.9***	58.1±6.0	28.2±9.5
*Viguiera tuberosa*	93.0±3.0*	74.5±1.9**	33.6±6.2	30.4±5.4
*Calyptocarpus biaristatus*	93.4±4.3*	39.2±6.1	48.2±2.9	21.7±4.3
*Hyalis argentea*	98.3±0.1****	33.3±12.4	32.9±4.5	9.8±9.5
*Helenium radiatum*	97.9±0.4***	3.7±2.1	97.9±0.4***	70.1±0.1*
*Gaillardia megapotamica*	98.3±0.2****	98.1±0.4****	98.1±0.1***	77.7±0.9***
*Verbesina subcordata*	84.7±3.1***	72.1±4.3*	81.3±2.8**	26.7±0.7
*Gymnocoronis spilanthoides*	90.0±2.9***	62.0±5.7*	89.6±0.2**	6.3±2.6
*Viguiera anchusaeifolia*	82.0±4.6**	1.9±0.5	51.8±4.5	9.3±8.0
*Thelesperma megapotamicum*	97.9±0.2**	95.6±1.1***	65.5±3.3	16.5±9.9
*Zexmenia buphtalmiflora*	96.8±1.7***	81.7±2.3***	64.4±1.0	21.6±5.9
*Vernonia plantaginoides*	92.2±1.3**	16.4±9.2	9.6±8.2	1.5±0.2

*p < 0.05, **p < 0.01 and ***p < 0.0005 and ****p < 0.0001.

## Conclusion

In this investigation, we have determined the trypanocidal activity of 13 Argentinean Asteraceae species. The results obtained in this research show the potential of Asteraceae species as a source of lead molecules for the development of new Chagas´disease chemotherapy. Fractionation of the most promising extracts and the isolation of the bioactive compounds will be carried out.
